# Comparative genomic analysis of five freshwater cyanophages and reference-guided metagenomic data mining

**DOI:** 10.1186/s40168-022-01324-w

**Published:** 2022-08-17

**Authors:** Kang Du, Feng Yang, Jun-Tao Zhang, Rong-Cheng Yu, Ziqing Deng, Wei-Fang Li, Yuxing Chen, Qiong Li, Cong-Zhao Zhou

**Affiliations:** 1 School of Life Sciences, University of Science and Technology of China, Hefei, 230027 Anhui China; 2 BGI-Shenzhen, Shenzhen, 518083 China; 3 BGI-Beijing, BGI-Shenzhen, Beijing, 100101 China

**Keywords:** Cyanobacterium, Freshwater cyanophage, Whole-genome sequencing, Reference genome, Metagenomics

## Abstract

**Background:**

As important producers using photosynthesis on Earth, cyanobacteria contribute to the oxygenation of atmosphere and the primary production of biosphere. However, due to the eutrophication of urban waterbodies and global warming, uncontrollable growth of cyanobacteria usually leads to the seasonal outbreak of cyanobacterial blooms. Cyanophages, a group of viruses that specifically infect and lyse cyanobacteria, are considered as potential environment-friendly agents to control the harmful blooms. Compared to the marine counterparts, only a few freshwater cyanophages have been isolated and genome sequenced to date, largely limiting their characterizations and applications.

**Results:**

Here, we isolated five freshwater cyanophages varying in tail morphology, termed Pam1~Pam5, all of which infect the cyanobacterium *Pseudanabaena mucicola* Chao 1806 that was isolated from the bloom-suffering Lake Chaohu in Anhui, China. The whole-genome sequencing showed that cyanophages Pam1~Pam5 all contain a dsDNA genome, varying in size from 36 to 142 Kb. Phylogenetic analyses suggested that Pam1~Pam5 possess different DNA packaging mechanisms and are evolutionarily distinct from each other. Notably, Pam1 and Pam5 have lysogeny-associated gene clusters, whereas Pam2 possesses 9 punctuated DNA segments identical to the CRISPR spacers in the host genome. Metagenomic data-based calculation of the relative abundance of Pam1~Pam5 at the Nanfei estuary towards the Lake Chaohu revealed that the short-tailed Pam1 and Pam5 account for the majority of the five cyanophages. Moreover, comparative analyses of the reference genomes of Pam1~Pam5 and previously reported cyanophages enabled us to identify three circular and seven linear contigs of virtual freshwater cyanophages from the metagenomic data of the Lake Chaohu.

**Conclusions:**

We propose a high-throughput strategy to systematically identify cyanophages based on the currently available metagenomic data and the very limited reference genomes of experimentally isolated cyanophages. This strategy could be applied to mine the complete or partial genomes of unculturable bacteriophages and viruses. Transformation of the synthesized whole genomes of these virtual phages/viruses to proper hosts will enable the rescue of *bona fide* viral particles and eventually enrich the library of microorganisms that exist on Earth.

Video abstract

**Supplementary Information:**

The online version contains supplementary material available at 10.1186/s40168-022-01324-w.

## Background

Cyanobacteria, also known as blue-green algae, are a class of ancient photoautotrophic prokaryotes [1], which have evolved on Earth for approximately 3.5 billion years [2]. They are widely distributed in various aquatic ecosystems, such as fresh, brackish, and marine waters, and also in some terrestrial habitats [3, 4]. In the past century, the accelerated urbanization and industrialization lead to eutrophication of the natural waterbodies and global warming, which allow the uncontrollable growth of cyanobacteria, and eventually cause the dense water blooms worldwide [5]. It was reported that bloom-forming cyanobacteria could reach a density as high as 10^7^~10^8^ cells per milliliter in the freshwater bodies [6, 7]; the dieback and decomposition of which could cause oxygen depletion, resulting in the mass death of demersal fish and other oxygen-sensitive animals [5, 8]. In addition, lysis of dense cyanobacteria usually leads to the release of a variety of toxins, which are harmful to the birds, mammals, and even humans around the waterbodies [9–11]. The outbreak of cyanobacterial blooms happens in 30~40% of the world’s lakes and drinking water reservoirs and more seriously up to ~80% of the freshwater bodies in developing countries such as China [12]. Thus, it is an urgent necessity to develop new methods to monitor the water blooms for the early warning and governance [13].

As reported, the bloom-forming cyanobacteria host diverse and abundant cyanophages, which could cause ~50% of global cyanobacterial mortality [14]. Cyanophages, as “the natural predators of cyanobacteria,” are a group of viruses that specifically infect cyanobacteria [15]. They are involved in regulating the abundance, community structure, and succession of cyanobacterial populations [16, 17]; and ultimately, play vital roles in the host-virus co-evolution, water quality preservation, and global biogeochemical cycling [18, 19]. The decrease of cyanobacterial biomass in the eutrophic lake is commonly accompanied with the infection of cyanophages [20], indicating that cyanophages might become potential environment-friendly agents to control the harmful cyanobacterial blooms [21].

Cyanophages usually contain a double-stranded DNA (dsDNA) genome, most belonging to the order of *Caudovirales*, which are generally classified into *Myoviridae*, *Siphoviridae*, and *Podoviridae* families according to the tail morphology [22, 23]. Although the first cyanophage—LPP-1 was isolated from a freshwater pond in 1963 [24], the majority of studies have been focused on the marine cyanophages to date. As shown in the Virus-Host database (https://www.genome.jp/virushostdb), 152 cyanophages have been genome sequenced, most of which were isolated from the marine cyanobacteria *Synechococcus* and *Prochlorococcus*. In contrast, to our knowledge, only 19 genome sequences of freshwater cyanophages have been reported, namely seven *Myoviridae* Ma-LMM01 [25], MaMV-DC [26], S-CRM01 [27], A-1(L) [28], N-1 [28], B3 [29], and B23 [29]; five *Siphoviridae* S-2L [30], S-LBS1 [31], CrV-01T [32], Mic1 [33], and Me-ZS1 [34]; and five *Podoviridae* PP [35], Pf-WMP3 [36], Pf-WMP4 [37], A-4(L) [38], and S-EIV1 [39], in addition to the two so-called tailless cyanophages PaV-LD [40] and PA-SR01 [41]. Despite metagenomic analysis showing that various aquatic environments are rich of cyanophages [42], the lack of well-characterized freshwater cyanophages strongly limited our knowledge on their evolution and application.

The Lake Chaohu, one of the five largest freshwater lakes in China, suffers from massive cyanobacterial blooms annually in the summer. Recently, we isolated and successfully cultured a new strain of cyanobacterium—*Pseudanabaena mucicola* Chao 1806, characterized by 16S rRNA combined with whole-genome sequencing. Using this strain as the host, we further screened and isolated five freshwater cyanophages of various tail morphologies from the Lake Chaohu: Pam1 and Pam5 with a short tail, Pam2 and Pam4 with a long tail, and Pam3 with a contractile tail. Whole-genome sequencing and comparative analyses showed that these five cyanophages possess distinct genome features and evolutionary relationships. The relative abundance of Pam1~Pam5 in the Lake Chaohu was calculated according to the reads from two metagenomic data. Moreover, based on the reference genomes of Pam1~Pam5 and previously reported cyanophages, ten virtual freshwater cyanophages have been mined from the metagenomic data.

## Methods

### Isolation and purification of cyanophages Pam1~Pam5

The water samples collected from 11 estuaries (Table S1) of the Lake Chaohu in September, 2018, were concentrated to about 100-fold by ultrafiltration and then applied to infect cyanobacterial strain *P. mucicola* Chao 1806 (Fig. S1). The double-layer plaque assays were performed at least three times to isolate cyanophages. The crude lysate was treated with 1 μg/mL DNase I and RNase at 37 °C for 1 h to digest the host nucleotides. Afterwards, NaCl was added to the solution at a final concentration of 0.5 M, followed by incubation at 4 °C for 2 h. The cyanophage particles were pooled by centrifugation (8000 g, 4 °C, 20 min) and then incubated with 10% polyethylene glycol 8000 at 4 °C for 10 h. After 10 min of centrifugation, the pellets were resuspended in 2 mL SM buffer (50 mM Tris, pH 7.5, 10 mM MgSO_4_, 100 mM NaCl). The viral particles were further purified by cesium chloride (CsCl) density gradient centrifugation (1.25 to 1.45 mg/mL, 100,000 g, 4 h, 4 °C). The corresponding phage band was collected by a syringe and dialyzed against SM buffer. Totally, five strains of cyanophages were isolated and purified, termed Pam1~Pam5 after their host, of which Pam1 and Pam5 are always coexisted.

After infecting *P. mucicola* Chao 1806, a single plaque on the agar plate (containing Pam1 and Pam5) was picked and cultured in BG11 liquid medium. PCR assays against this crude lysate were performed using primers designed against the *terL* genes of Pam1 and Pam5, respectively: the primers of Pam1: 5′-CAGAAGGAGCTGGCAGCGAGGCAA-3′ (forward) and 5′-ACAATATCCCGTCGCCGTTCGCTG-3′ (reverse) and the primers of Pam5: 5′-CTCGATTGCCCTCTCTCTGAGGTG-3′ (forward) and 5′-CGAATGACGACCTTTGGCCCTTGC-3′ (reverse).

### Negative-stain transmission electron microscopy

To characterize the phage morphology, a drop of 3.5 μL sample containing purified cyanophages was layered onto a hydrophilized carbon-coated copper grid and incubated with 2% uranyl acetate for 1 min. The negatively stained particles were examined with a Tecnai G2 Spirit BioTWIN 120-kV transmission electron microscope (FEI Company, Hillsboro, USA).

### Genomic DNA extraction and sequencing

Equal volume of 2× lysis buffer (final concentration of 20 mM EDTA and 0.5% SDS) was added to the SM buffer with cyanophages, which was then incubated with 50 μg/mL proteinase K at 56 °C for 1 h. The phage suspension was sequentially treated with phenol, phenol-chloroform-isoamyl alcohol (25:24:1), and chloroform at a volume ratio of 1:1, respectively. Then, the genomic DNA was precipitated with 1/10 volume of 3 mol/L CH_3_COONa, pH 7.5, followed by threefold volume of ethanol at −80 °C for 4 h. The precipitated phage genomic DNA was washed twice with 70% ethanol and then resuspended with sterile ultrapure water at an appropriate volume. Subsequently, the extracted genomic DNA was sequenced by whole-genome shotgun (WGS) strategy to construct a library of different inserts, based on the MGISEQ-2000 platform (BGI-Shenzhen, China) or Illumina NovaSeq platform (Shanghai Personal Biotechnology Co., Ltd., China). For each cyanophage, after removing the adapters and poor-quality reads, all the clean reads were applied for genome assembly with the software SPAdes [43] to obtain the de novo assembled contigs.

### Genome annotation and characterization

The open reading frames (ORFs) were predicted by GeneMarkS (http://exon.gatech.edu/GeneMark/genemarks.cgi) and Glimmer (http://ccb.jhu.edu/software/glimmer) implemented in DNA Master v5.23 (http://cobamide2.bio.pitt.edu). Using BLASTp program v1.12.0 [44], the translated ORFs were searched against the nr protein database (released on 2021 July 16) in NCBI (https://www.ncbi.nlm.nih.gov/), and the results with *e*-values < ~10^−3^ are considered believable. For each encoded protein, hit with the minimal *e*-value is regarded as ortholog. Alternatively, HHpred analysis [45] against pfamA v35 [46] and conserved domain database v3.18 [47] were carried out with the default parameters (1e^−3^ of *e*-value cutoff for MSA generation), respectively, for annotation of the ORFs. Operons of the genome were predicted by operon-mapper [48], whereas tRNAs were found by Aragorn [49] with both strands. Moreover, the genome map was drawn with CGView (http://cgview.ca) and Proksee (https://proksee.ca), whereas the proteomic trees and genome alignments of phages were conducted using ViPTree v3.0 [50] against the dsDNA reference viruses. The phylogenetic trees were made by MEGA program v10.2 [51], using ClustalW for multiple sequence alignment together with maximum-likelihood method for reconstructing branches. The CRISPR-Cas prediction of host genome was fulfilled by CRISPR-Cas Finder of CRISPR-Cas++ v1.1.2 [52] with Cas gene detection; moreover, spacers of host were aligned with phage genomic DNA via BLASTn v2.12.0 [44] and MultAlin [53]. Genome alignment showed that Pam2 shares several DNA segments identical to the spacer regions of a CRISPR-Cas system in *P. mucicola* Chao 1806, suggesting that there should be an anti-CRISPR system in Pam2. Thus, the anti-CRISPRs of Pam2 were predicted by AcrHub (https://pacrispr.erc.monash.edu/AcrHub/) against PaCRISPR v1.2 and AcRanker, in combination with the predication of Acr-associated (Aca) proteins via BLASTp v1.12.0.

### Viral metagenomic sequencing

The 5 L water samples were collected from Nanfei estuary in the Lake Chaohu in October, 2017, and June, 2021, respectively. After sequentially filtering with 5, 2, 1.2, 0.8, and 0.45 μm filter membrane, most of the bacteria in the water sample were removed. Then, the filtered water sample was treated with FeCl_3_ to precipitate the phages, which were then resuspended in oxalic acid buffer. DNase I and RNase were added to the suspension to remove free nucleic acids in the viral concentrates. Subsequently, mixed genomes of the various precipitated phages were extracted in the same manner as those for cyanophages Pam1~Pam5 and further applied to viral metagenomic sequencing with Illumina NovaSeq platform (Shanghai Personal Biotechnology Co., Ltd., China).

### Metagenomic fragments recruitment

The recruitment of metagenomic fragments against the sequencing data of Nanfei estuary was performed using a reciprocal best-hit BLAST (RBB) strategy as previously reported [54]. By the program BLAST+ v1.12.0 [44], all the sequencing reads were collected and built as a nucleotide database, which was then compared with the sequences of proteins encoded by cyanophages Pam1~Pam5, respectively, using tBLASTn [44] with the parameters of −max_target_seqs = 1,000,000, −outfmt = 6, −seg = no, and −evalue = 10^−3^. Afterwards, the hits corresponding to each Pam phage were extracted, respectively, and moreover, they were searched against the database containing all encoded proteins of the tailed phages by program BLASTx v1.12.0 [44] with the parameters of −max_target_seqs = 1, −evalue = 10^−3^. Based on the comparison results, the fragments that best match cyanophages Pam1~Pam5 were extracted from the metagenomic sequencing data, respectively, and seemed as hits of each Pam phage. The relative abundance of each Pam phage against all the five phages was calculated, and the genome sizes of Pam phages were usually used as normalized standard.

### Assembly of metagenomic fragments and identification of the phage contigs

FastQC v0.11.9 (https://www.bioinformatics.babraham.ac.uk/projects/fastqc) was employed for quality control on the raw metagenomic sequencing data of Nanfei estuary, which was then trimmed by Trimmomatic v0.39 [55] to remove adapters and low-quality reads. The obtained clean reads were first assembled by MEGAHIT v1.2.9 [56] to acquire various contigs. These putative contigs were further assessed by VirSorter2 v2.1 [57] to identify the phage ones. Afterwards, the potential phage contigs between 10 and 200 kb were annotated by Prodigal v2.6.3 [58] and then clustered with *Caudovirales* (tailed bacteriophages) that is deposited in the Virus-Host database (https://www.genome.jp/virushostdb) in addition to cyanophages Pam1~Pam5 via ViPTree v3.0 [50]. Besides, comparative genome analyses were performed by BLAST+ v1.12.0 [44] in combination with Mauve v2.4.0 [59].

## Results

### Isolation and morphology of cyanophages infecting *P. mucicola* Chao 1806

**Fig. 1:**
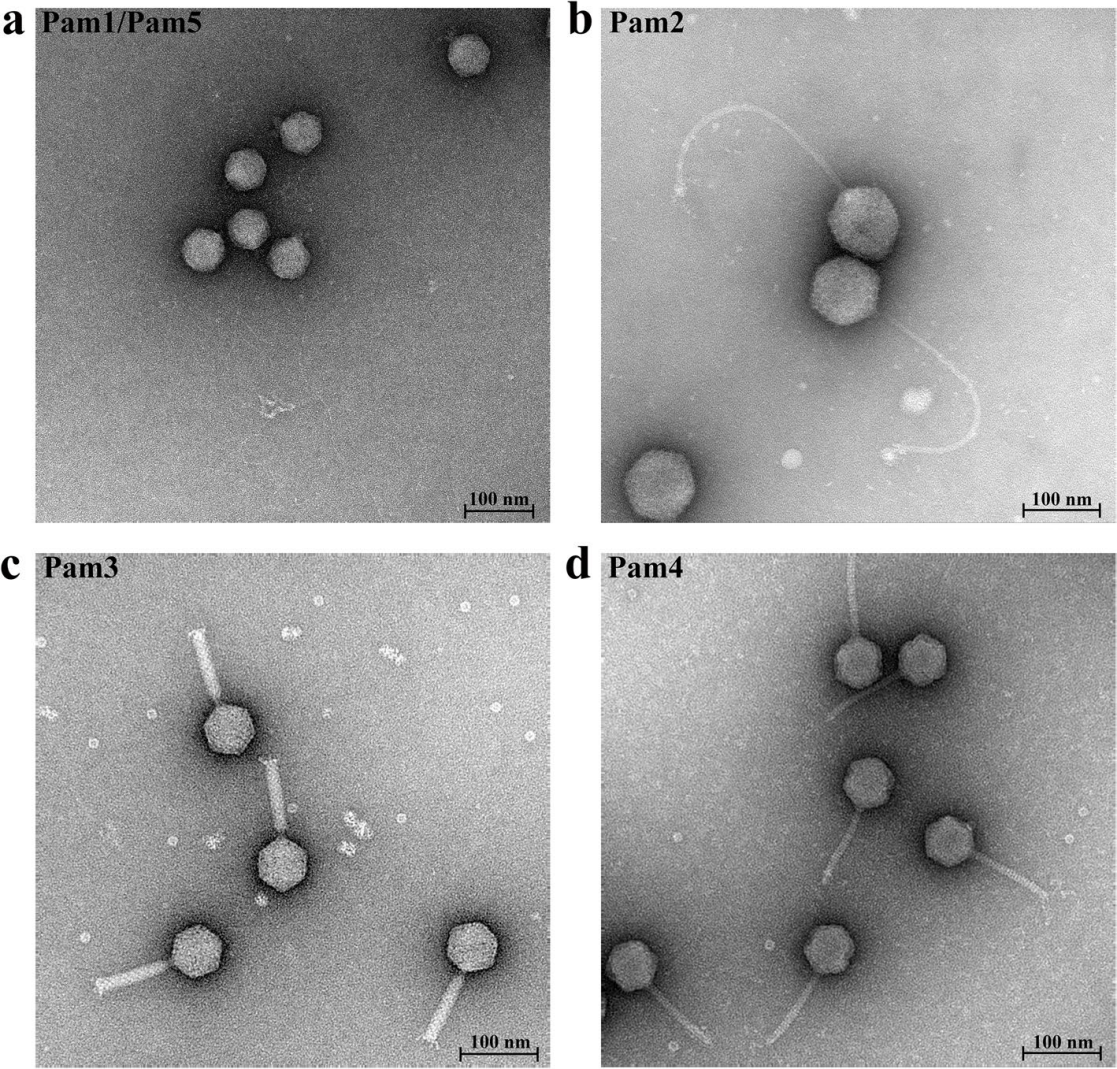
The morphologies of Pam1~Pam5 particles. **a ***Podoviridae* Pam1 possesses an icosahedral head of ~65 nm in diameter, in addition to a short tail of ~40 nm in length. Pam5, found by genome sequencing, has an indistinguishable morphology with Pam1. **b ***Siphoviridae* Pam2 adopts an icosahedral head of ~100 nm in diameter and a long but noncontractile tail of ~300 nm in length. **c ***Myoviridae* Pam3 comprises an icosahedral head with a diameter of ~68 nm, followed by an ~110-nm-long and contractile tail. **d** *Siphoviridae* Pam4 has an icosahedral head of ~63 nm in diameter and a long tail of ~110 nm in length. The scale bar is 100 nm

Using *P. mucicola* Chao 1806 as the host, we isolated and purified four colonies of cyanophages from different water samples of the Lake Chaohu, which are sequentially termed Pam1~Pam4. To identify the morphology of Pam1~Pam4, we further applied these purified cyanophage particles to negative-stain transmission electron microscopy. The results showed that Pam1~Pam4 adopt three various tail morphologies (Fig. 1): *Podoviridae* Pam1, *Siphoviridae* Pam2 and Pam4, and *Myoviridae* Pam3. In detail, Pam1 possesses an icosahedral head of ~65 nm in diameter, in addition to a short tail of ~40 nm in length (Fig. 1a), whereas Pam3 comprises an ~68-nm head in diameter, followed by an ~110-nm-long contractile tail (Fig. 1c). Although Pam2 and Pam4 belong to the same family, they differ a lot from each other in morphology. Pam2 adopts an icosahedral head of ~100 nm in diameter and a long but noncontractile tail of ~300 nm in length (Fig. 1b), which are ~37 nm larger and ~190 nm longer compared to those of Pam4, respectively (Fig. 1d). Moreover, the host-range assays against about a dozen of cyanobacterial strains [60] isolated from the Lake Chaohu showed that cyanophages Pam1~Pam4 could only infect and lyse *P. mucicola* Chao 1806, suggesting their high host specificity. However, why they recognize and infect the same host remains elusive.

### Genome sequences of cyanophages Pam1~Pam5

**Fig. 2:**
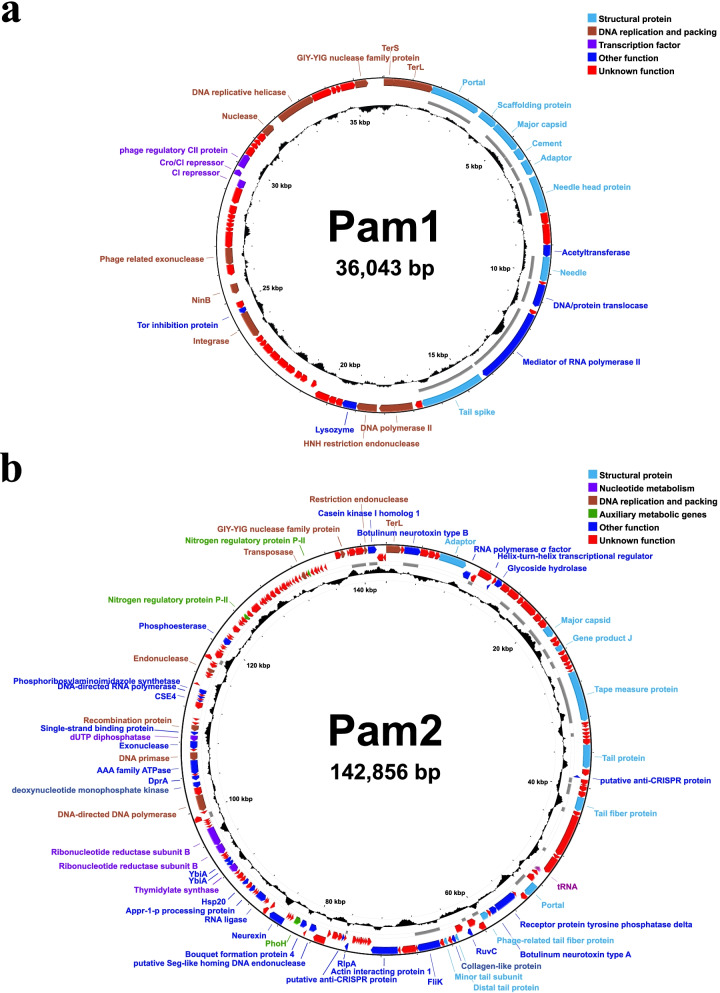
The circular genomic maps of cyanophages **a** Pam1 and **b** Pam2, respectively. Circles from the outmost to the innermost represent the following: predicted ORFs with known functions on (i) forward strand and (ii) reverse strand are labeled and colored based on their functions, (iii) structural proteins that identified by mass spectrometry are shown by gray lines, and (iv) G + C content plotted relative to the genomic mean of 35% G + C

From the whole-genome sequencing data of four colonies, we found that two circular genomes could be assembled in the Pam1 colony. It suggested that this colony contains two strains of cyanophages similar in morphology (Fig. 1a), which were termed Pam1 and Pam5, respectively. In fact, PCR assays indicated that Pam1 and Pam5 tend to coexist in the lysate of a single plaque of infection (Fig. S2). It showed that all the five strains of cyanophages Pam1~Pam5 possess a dsDNA genome, in length of 36,043, 142,856, 54,544, 48,349, and 39,509 bp, respectively (Fig. 2 and Fig. S3). Notably, the genome of Pam4, which possesses a G + C content up to 73.6% that contains the essential genes, is only about half or less to the previously reported genomes of long-tailed freshwater cyanophages [61].

GeneMarkS (http://exon.gatech.edu/GeneMark/genemarks.cgi) and Glimmer (http://ccb.jhu.edu/software/glimmer) analyses indicated that Pam1~Pam5 contain 60, 195, 72, 73, and 68 ORFs, respectively (Table S2). However, less than half of the ORFs could be annotated with a known function, except for ~73% of Pam3 ORFs. Based on BLASTp [44] and HHpred [45], the annotated proteins of Pam1, Pam4, and Pam5 are classified into four groups: structural protein, DNA replication and packing, transcription factor, and other function, whereas Pam2 and Pam3 possess more nucleotide metabolism and host-derived auxiliary metabolic genes (Fig. 2 and Fig. S3).

Similar to those previously reported phages, the structural genes of Pam1, Pam3, and Pam5 could be divided into two groups, head and tail genes, immediately following the terminase small (*terS*) and large (*terL*) subunit genes (Fig. 2 and Fig. S3). Thanks to our recently reported structure of Pam1 [62], the structural proteins for Pam1 and Pam5 could be better annotated, such as head proteins: portal, scaffolding, capsid, and cement protein, followed by tail proteins: adaptor, needle head, spike, needle, etc. (Fig. 2a and Fig. S3c). Besides several head proteins resembling those of the *Podoviridae* Pam1 and Pam5, the *Myoviridae* Pam3 consists of more tail proteins, including the stopper, sheath, tube, tape measure, tube initiator, fiber, and baseplate-related proteins (Fig. S3a). In contrast, the head and tail genes of *Siphoviridae* Pam2 or Pam4 are not sequentially aligned in successive gene clusters but interrupted by genes of distinct and/or unknown function (Fig. 2b and Fig. S3b). Compared to extensive studies on tail structures of *Podoviradae* and *Myoviradae* [63–65], the yet unknown tail structure of *Siphoviridae* makes it difficult to annotate the related genes.

### The phylogenetic analyses of Pam1~Pam5

**Fig. 3:**
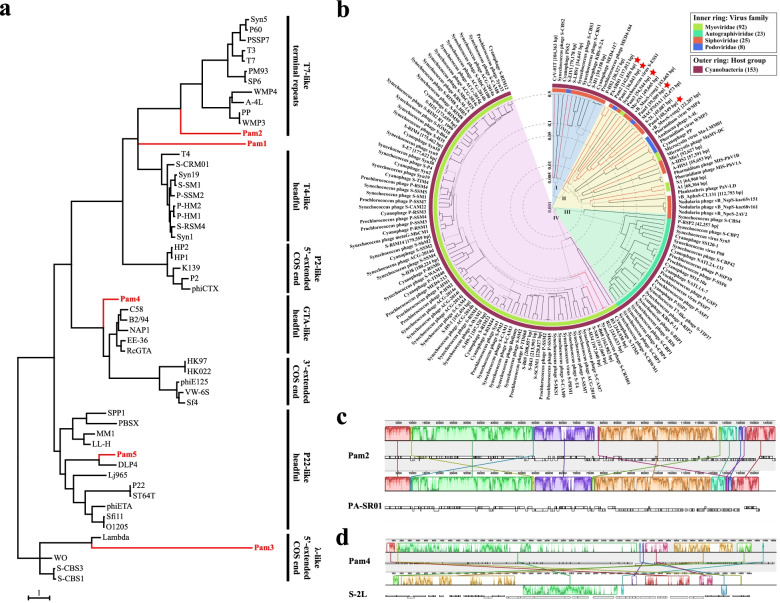
The phylogenetic analyses of cyanophages Pam1~Pam5. **a** Maximum-likelihood phylogenetic tree of TerL proteins from different phages indicates the putative DNA packaging mechanisms of Pam1~Pam5. Vertical lines cluster the phages that have similar DNA termini. The TerL sequences, except those of Pam1~Pam5, were classified as described previously [33, 67]. The bootstrap analysis was performed with 1000 repetitions, and the scale bar represents the number of substitutions per site. Cyanophages Pam1~Pam5 are highlighted in red. **b** The proteomic tree of Pam1~Pam5 against 148 previously reported cyanophages. The genome sequences of 19 freshwater cyanophages are colored in red. The four clusters are labeled in different colors: (I) marine *Siphoviridae* (blue), (II) freshwater cyanophages (yellow), (III) marine *Autographiviridae* (green), (IV) marine *Myoviridae* (pink). Pam1~Pam5 are marked with a red star, respectively. **c**, **d** Whole-genome alignments of **c** Pam2 against PA-SR01 (GenBank accession number: MT234670.1) and **d** Pam4 against S-2L (GenBank accession number: MW334946.1), respectively. The alignments were performed with the software Mauve [59]. Blocks with the same color indicate the homologous regions of two genomes, which are also connected by the same color lines. The height of the similarity profile corresponds to the sequence similarity, whereas regions outside the colored blocks indicate the lack of homology among the two genomes. The inverted blocks are indicated below the genome’s center axis (a horizontal line). The numbers above the alignments indicate the nucleotide positions in the genome

The phylogenetic analysis of TerL, which cuts the viral genome and fuels DNA translocation [66], could classify the tailed dsDNA phages into seven groups of DNA packaging mechanism [67]. It showed that Pam2 falls into the “T7-like terminal repeats” group, whereas Pam3, Pam4, and Pam5 are classified into the groups of “*λ*-like 5′-extended COS end”, “GTA-like headful”, and “P22-like headful”, respectively, leaving Pam1 unclustered (Fig. 3a). Despite infecting a same strain of cyanobacterial host, Pam1~Pam5 might use distinct mechanisms to fulfill the packaging of genome.

Moreover, to explore the evolutionary relationships of Pam1~Pam5 with other previously reported cyanophages, ViPTree [50] was used to build the proteomic tree based on the genomes of 115 completely sequenced cyanophages from Virus-Host database (excluding 37 redundant genomes), in addition to the genomes of 33 cyanophages that are not included in the Virus-Host database, but deposited to the NCBI nucleotide database (https://www.ncbi.nlm.nih.gov/nuccore). According to the normalized genome similarity scores (SG), most of the freshwater cyanophages, including Pam1~Pam5, fall into cluster II, whereas marine cyanophages belong to cluster I/*Siphoviridae*, III/*Autographiviridae*, or IV/*Myoviridae*, respectively (Fig. 3b).

As the most distinct one from the other four cyanophages, Pam2 shares a very close evolutionary position (0.1 < SG < 0.5) with PA-SR01 (Fig. 3b), a strain of freshwater *Pseudanabaena* cyanophage isolated from a reservoir in Singapore [41]. BLASTn [44] analysis showed that Pam2 has a sequence identity of 94.69% over 57% PA-SR01 genome, and moreover, the whole-genome alignment revealed that these two phages share a very high homology in most ORFs, which are clustered in six collinear regions (Fig. 3c). This large-scale genomic collinearity suggested they most likely share a common ancestor, despite isolated from two distant waterbodies.

Pam4 shares a relatively close evolutionary distance, at the same branch of 0.05 < SG < 0.1, with S-2L (Fig. 3b), a strain of freshwater *Synechococcus* cyanophage isolated from a waterbody in Leningrad [30]. Comparison of the whole genome showed that though genome of Pam4 has high synteny with that of S-2L, multiple gene rearrangements, inversions, and deletions could be observed (Fig. 3d). In contrast, Pam1, Pam3, and Pam5 have low similarities with the bacteriophages that are located at the adjacent branches, with an SG value of < 0.05 in the proteomic tree (Fig. 3b). Notably, Pam1 defines a unique branch with an SG value of 0.005, compared to Pam3~Pam5.

Although infecting the same host *P. mucicola* Chao 1806, the five cyanophages Pam1~Pam5 seem to be distinct from each other at the viewpoint of evolution. It might be due to the lack of enough reference genomes of freshwater cyanophages, which are needed for the characterization of more general features.

### The *Podoviridae* Pam1 and Pam5 possess lysogeny-associated genes

**Fig. 4:**
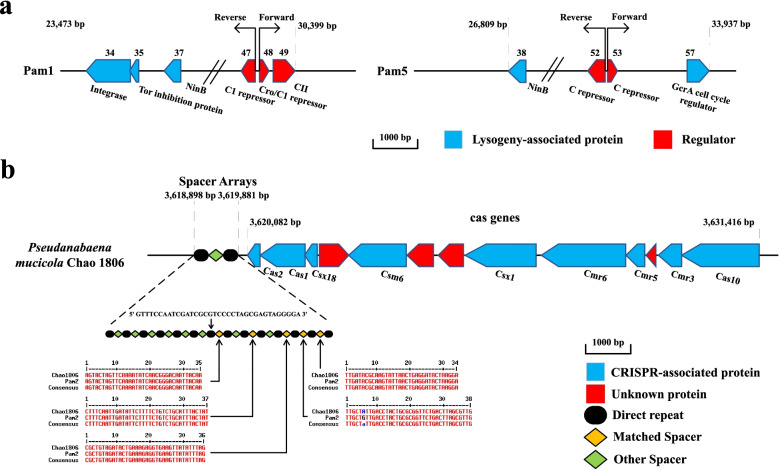
Genomic analyses of cyanophages Pam1, Pam5, and Pam2. **a** Schematic diagram of the organizations of Pam1 and Pam5 lysogeny-associated genes. The genes that encode transcription regulators are colored in red, whereas other lysogeny-associated genes are colored in blue. The directions of transcription are indicated by arrows. **b** Pam2 possesses several nearly identical DNA segments with the host CRISPR spacers. Multalin alignments were performed between the spacers of host *P. mucicola* Chao 1806 and DNA segments of Pam2 genome

Despite sharing a nearly identical morphology and usually coexisting in a same plaque, Pam1 and Pam5 differ a lot in genomic sequence (Fig. S4). Nevertheless, both Pam1 and Pam5 contain a couple of genes that encode transcription factors (Fig. 2a and Fig. S3c), especially the putative repressor or regulatory genes: gp47~gp49 of Pam1 and gp52~gp53 of Pam5 (Fig. 4a), which might be associated with the regulation of lysogenic-lytic cycle.

AlphaFold 2.0 prediction [68] combined with structural superposition showed that gp47~gp49 of Pam1 possess a core structure similar to the regulators CI, Cro, and CII of phage λ [69], whereas gp52~gp53 of Pam5 are structural homologs of C and Cox of phage P2 [70], respectively (Fig. S5). In fact, gp53 of Pam5 highly resembles the C repressor than Cox (Fig. S5), suggesting a same function but different DNA-binding patterns during the induction of lysis. These regulators all display a helix-turn-helix structure, especially with a recognition helix that possesses basic residues responsible for interacting with the major groove of DNA (Fig. S5). Moreover, both Pam1 and Pam5 genomes possess two transcriptional directions, which are converted between gp47 and gp48 of Pam1 and gp52 and gp53 of Pam5 (Fig. 4a), similar to CI/Cro in phage *λ* and C/Cox in phage P2, respectively. Despite the gp34 of Pam1 was assigned to an integrase, we failed in identifying a CIII in Pam1 or an integrase in Pam5.

Furthermore, Pam1-gp37 and Pam5-gp38 are annotated as NinB (Fig. 4a), which participates in the recombination of *λ*-like phages via binding to the single-stranded DNA [71]. For Pam1, NinB/gp37 might function with the integrase gp34 to insert the genome of Pam1 into host genome during the lysogenic cycle under adverse environments. In the lytic cycle, Pam1 might utilize the Tor inhibition protein TorI/gp35 (Fig. 4a), a homolog of phage-encoded excisionase ^λ^Xis [72], to excise Pam1 genome from the host genome and prevent the reintegration of Pam1 genome. Besides, gp57 of Pam5 was annotated as cell cycle regulator GcrA, which might play a similar role in the lysogeny-lysis switch of cyanophages as that in the cell cycle progression of α-proteobacteria: directly interacting with RNA polymerase for the global regulation of gene expression [73].

Altogether, both Pam1 and Pam5 possess lysogeny-associated genes, indicating a putative lysogenic cycle in *P. mucicola* Chao 1806. The similar lysogeny-associated genes, and even a similar life cycle after infection, might make Pam1 and Pam5 tend to coexist in a same colony.

### The *Siphoviridae* Pam2 possesses CRISPR spacers and additional tRNAs

The *Siphoviridae* Pam2 has the longest genome length among the five cyanophages, corresponding to the largest head size of viral particles (Figs. 1 and 2). Pam2 also possesses more ORFs, most of which were annotated to “hypothetical proteins”. Besides the necessary genes for DNA replication and phage assembly, the other genes, especially those of unknown function, might contribute to the interplay between Pam2 and its host.

The genome alignment showed that Pam2 shares several DNA segments identical to its host *P. mucicola* Chao 1806 (Fig. 4b). Further analysis of CRISPR-Cas (https://crisprcas.i2bc.paris-saclay.fr/CrisprCasFinder/Index) revealed that these segments were predicted as part of the spacers in the region from 3,618,898 to 3,631,416 bp on the host genome that encodes a putative type III CRISPR-Cas system (Fig. 4b). This CRISPR-Cas system contains several CRISPR-associated genes—the *cas* genes, in addition to the CRISPR arrays: 14 direct repeat sequences interspersed by 13 spacers (Fig. 4b). Notably, sequences identical to the 7th, 9th, 11th, and 13th spacers could be found in Pam2 genome, whereas the 12th spacer has a single mismatch with a 38-bp DNA segment of Pam2 genome (Fig. 4b). In addition, we found four more segments in Pam2 genome, which are identical to the sequences distributed in the other three spacer regions of the host genome (Fig. S6). As we know, CRISPR-Cas is an adaptive immune system that could protect bacteria and archaea from virus and plasmid infection, the spacer region of which is always acquired from the protospacer—invading DNA segment from virus or plasmid [74, 75]. It suggested that, among the five Pam cyanophages, Pam2 and its host *P. mucicola* Chao 1806 have adopted a CRISPR and anti-CRISPR mechanism along the long history of co-evolution. In fact, two putative anti-CRISPRs (gp38 and gp80) could be predicted based on the sequence analyses of Pam2 genome.

Different from the other four cyanophages, Pam2 possesses four tRNA genes, namely tRNA^Arg^, tRNA^Lys^, tRNA^Asn^, and tRNA^Gly^ (Table S3). These additional tRNAs might enable the efficient translation of Pam2 proteins during the amplification of progeny phages in the host. In fact, the four residues Arg, Lys, Asn, and Gly are rich in the structural proteins of Pam2, up to 24%, 23.3%, and 27% in the major capsid protein, portal protein, and tape measure protein, respectively.

### The RBPs that target the extracellular polysaccharides of host cell surface

Except for a report on the tail protein ORF36 of *Myoviradae* A-1(L) that recognizes the lipopolysaccharides during infecting *Anabaena* sp. PCC 7120 [76], the specific interaction between cyanophage and host remains largely unknown. A couple of ORFs in Pam1, 2, 3, and 5 genomes have been annotated as the putative receptor-binding proteins (RBPs), which possess a primary sequence similarity less than 20% to each other (Table S4). Structural analysis of Pam1 tailspike gp17 (PDB code of 7eea) revealed a C-terminal receptor-binding domain that contains a right-handed parallel β-helix and a distal β-sandwich, both of which are proposed to be responsible for the recognition of host extracellular polysaccharides [62]. Moreover, we predicted the 3-D structures of putative RBPs of Pam2, 3, and 5 via AlphaFold 2.0 [68]. It showed that both tail fibers, gp43 of Pam2 and gp24 of Pam3, possess a deformed β-sandwich motif of two-layered β sheets at the C-terminus (Fig. S7), sharing a root-mean-square deviation (RMSD) of 4.5 Å over 50 Cα atoms and 3.5 Å over 61 Cα atoms with the β-sandwich motif of Pam1-gp17, respectively. The putative RBP gp17 of Pam5 also adopts a β-sandwich fold (Fig. S7), with an RMSD of 2.8 Å over 60 Cα atoms to Pam1-gp17. DALI search [77] revealed that all these RBPs are somewhat structurally similar to polysaccharide-binding proteins, such as glycoside hydrolase, exopolysaccharide biosynthesis protein, or cell surface glycoprotein (Table S5). It suggested that these putative RBPs could also target the extracellular polysaccharides at the cell surface of host *P. mucicola* Chao 1806 to initiate the infection, despite recognizing various sugar units.

### The predicted abundance of Pam1~Pam5 in the Lake Chaohu based on the metagenomic data

In order to assess the relative abundance of cyanophages Pam1~Pam5 in the Lake Chaohu, we performed metagenomic fragments recruitment analyses using the sequencing data of water sample collected at the Nanfei estuary to the Lake Chaohu in October, 2017. The reads that match each ORF of cyanophages Pam1~Pam5 at a 30~100% amino acids identity were recruited and mapped to the corresponding positions of each Pam genome (Fig. S8). It suggested that all the five cyanophages Pam1~Pam5 exist but in various abundances.

**Fig. 5:**
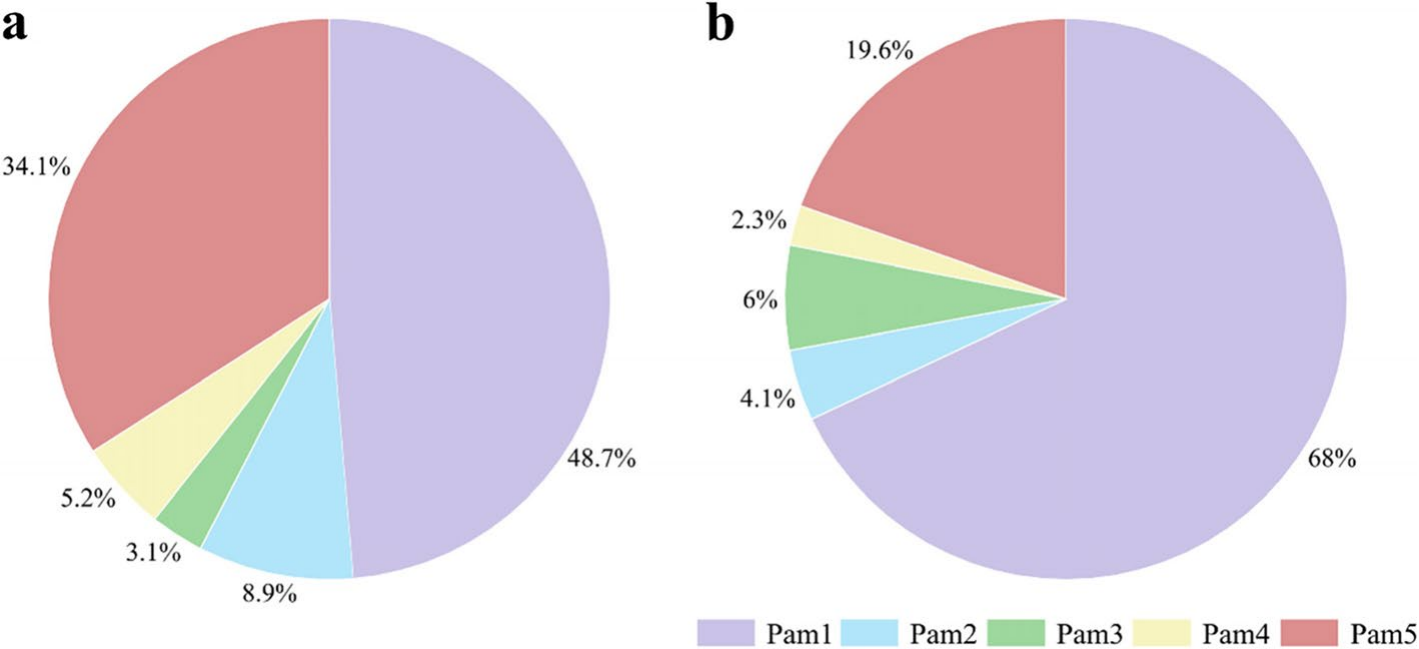
The relative abundance of the five cyanophages Pam1~Pam5 in the Lake Chaohu. The percentages were calculated according to metagenomic data of two water samples obtained at the Nanfei estuary in **a** October, 2017, and **b** June, 2021, respectively. The abundance (number of reads) was normalized to the total number of genome length of each cyanophage

Moreover, the relative abundance of Pam1~Pam5 was calculated. The results showed that the abundance of *Podoviridae* Pam1 and Pam5 account for up to 82.8% (48.7% Pam1 and 34.1% Pam5), followed by *Siphoviridae* Pam2 and Pam4 with an abundance of 8.9% and 5.2%, respectively, and *Myoviridae* Pam3 of only 3.1% (Fig. 5a). In addition, the results of metagenomic analyses against the sequencing data of water sample collected at the same place in June, 2021, revealed that *Podoviridae* Pam1 and Pam5 have the abundance as high as 87.6%, of which Pam1 is the most abundant one up to 68% (Fig. 5b). The abundance of *Siphoviridae* Pam2 and Pam4 decreases to 4.1% and 2.3%, respectively, whereas that of *Myoviridae* Pam3 slightly increases to 6% (Fig. 5b). It suggested that though the relative abundance of Pam1~Pam5 in the Lake Chaohu changes in different seasons, the short-tailed Pam1 and Pam5 always account for the majority of these five cyanophages.

The different abundances in the natural freshwater environments indicated that the five cyanophages Pam1~Pam5 might compete against each other during the lytic cycle and possess distinct capacities to infect the same host. The putative lysogenic-lytic cycle of Pam1 and Pam5, which help them better adapt to diverse environments, in addition to the relatively smaller size of genome, might contribute to their dominance among the five cyanophages in the Lake Chaohu.

### Mining of three circular and seven linear cyanophage genomes from the metagenomic data

In-depth recruitment analyses of the metagenomic data of October, 2017, revealed that, besides the reads that best match the cyanophages Pam1~Pam5, there are tens of millions of reads that resemble other known tailed dsDNA phages, which were assembled into thousands of contigs at different lengths. According to the popular genome length of classic *Caudovirales* [78], 1235 contigs ranging from 10 to 200 kb were picked for further analyses. Finally, 98 putative metagenomic phage contigs were characterized by VirSorter2 [57], in which seven contigs with overlapped sequences could be assembled into circular genomes (Table S6, Fig. S9).

**Fig. 6:**
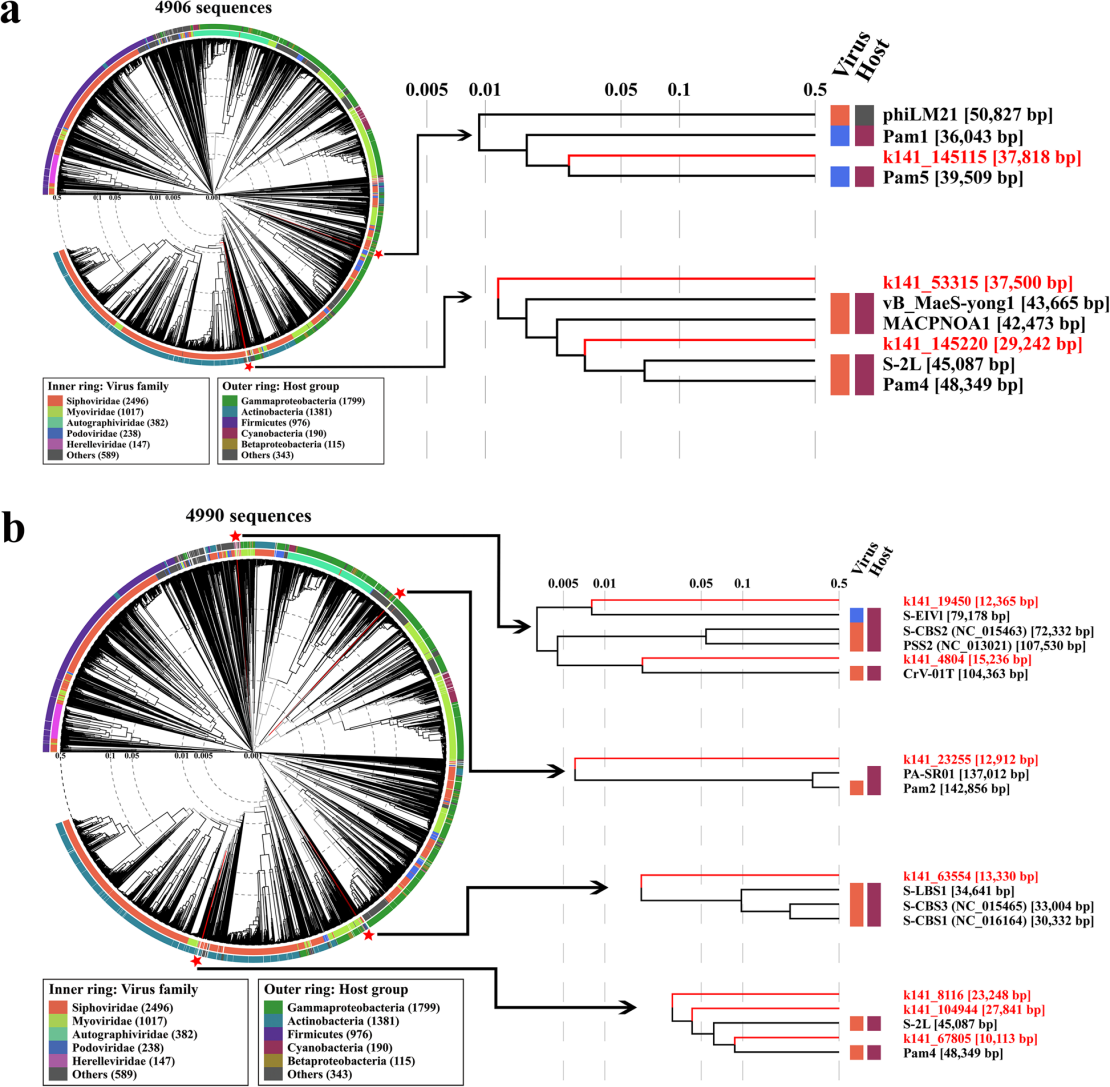
Phylogenetic analyses of the putative metagenome-assembled contigs. The proteomic tree of **a** seven circular contigs and **b** 91 linear contigs against 4894 tailed dsDNA phages plus our sequenced Pam1~Pam5, respectively. The 4894 tailed dsDNA phages include 185 cyanophages, of which 152 are from the Virus-Host database (including the redundant genomes) and 33 from the NCBI nucleotide database. The cyanophage clusters containing the metagenome-assembled contigs are labeled with a red star and then enlarged as insets. The tree was generated by the ViPTree server. Branch lengths were logarithmically scaled from the root of the entire proteomic tree

These seven circular contigs were further applied to construct the proteomic tree based on the reference genomes of 4894 tailed dsDNA phages (including 185 cyanophages) in addition to our sequenced Pam1~Pam5, to discover the putative cyanophage contigs. It showed that three out of seven circular contigs are clustered in the evolutionary branches with the known cyanophages, respectively (Fig. 6a). The contig k141_145115 falls into the group of Pam1 and Pam5, whereas contig k141_53315 and k141_145220 are clustered with Pam4, S-2L, MACPNOA1, and vB_MaeS-yong1 (GenBank accession numbers: KY697807.1 and MT855965.1). It suggested that these three circular contigs represent the genomes of cyanophages that have not yet been experimentally isolated.

The whole-genome alignments were performed to compare these three virtual cyanophages with their neighbors to validate the evolutionary relationships. Although Pam1 and Pam5 have low sequence similarity to each other (Fig. S4), it revealed that contig k141_145115 shares a somewhat high similarity with several regions of Pam1 and Pam5 genomes (Fig. S10a), respectively. Considering that contig k141_145115 closely resembles Pam5 at evolutionary level (Fig. 6a), it most likely infects the same host *P. mucicola* Chao 1806. The contigs k141_145220 and k141_53315 possess more homologous regions with the genome of Pam4, compared to the other three cyanophages (Fig. S10a).

Beyond the seven circular ones out of 98 putative phage contigs, the 91 linear contigs were also applied to phylogenetic analysis against 4899 reference phage genomes. It revealed that seven linear contigs could be clustered with the previously identified cyanophages: S-EIV1 [39], CrV-01T [32], PA-SR01 [41], S-LBS1 [31], S-CBS3 [67], S-CBS1 [67], and S-2L [30], in addition to Pam2 and Pam4 (Fig. 6b). It suggested that these contigs might be genomic fragments of putative cyanophages. Further whole-genome alignments showed that compared to the contigs k141_104944 and k141_67805 that possess somewhat higher similarity with the two reference genomes (S-2L and Pam4), respectively, the contig k141_8116 shares a pretty low sequence similarity (Fig. S10b), in accordance with the evolutionary distance in the above proteomic tree (Fig. 6b).

The proteomic and comparative genomic analyses proposed that the three circular and seven linear contigs assembled from the metagenomic data of the Lake Chaohu are probably complete and partial genomes of virtual cyanophages, respectively. Further host identification and cyanophage amplification are needed to validate these not-yet-culturable cyanophages.

## Discussion

To date, compared to hundreds of marine cyanophages, the freshwater cyanophages have been poorly identified. The Lake Chaohu, which suffers from dense cyanobacterial blooms from June to September every year, provides an ideal source for isolating and characterizing freshwater cyanobacteria and their symbiotic cyanophages. Using the host *P. mucicola* Chao 1806, we successfully isolated and sequenced five strains of freshwater cyanophages, termed Pam1~Pam5. These experimentally isolated cyanophages constitutes the seeds for establishing a library of freshwater cyanophages, which might be applied to the early warning and environment-friendly control of algal blooms.

It is known that the vast majority of bacteria are not culturable [79]; however, the high-throughput sequencing technology combined with bioinformatics analysis methods enabled us to recruit a large number of complete genomes, the so-called metagenome-assembled genomes of not-yet-cultured bacteria or phages [80–82]. Due to the high diversity and lack of conserved marker genes, the taxonomy of phages remains a big challenge. Despite the terminase, head proteins, or tail proteins have been used to characterize tailed phages, it is hard to distinguish cyanophages from bacteriophages, especially those without obvious cyanobacterium-derived auxiliary metabolic genes. Using the genomes of cyanophages Pam1~Pam5, together with 4894 previously sequenced genomes of tailed dsDNA phages, as the reference, we successfully constructed complete or partial genomes of ten virtual freshwater cyanophages from the metagenomic data of the Nanfei estuary at the Lake Chaohu (Fig. 6). Further iterated data mining, in combination with the growing metagenomic data and rationally designed experimental validations, will enable us to efficiently identify more freshwater cyanophages and enrich the library.

To validate these virtual cyanophages, it is vital to identify the host cyanobacteria. The most common approach for the prediction of host is to search identical DNA segments, which are originally acquired from the phages and become CRISPR spacers of host [83], in the phage and bacterial genomes. In 2019, Morimoto and colleagues identified 15 viral contigs belonging to cyanophages from metagenomic data on the basis of their *Microcystis* protospacers using CRISPR spacer-based host prediction [84]. Unfortunately, we failed in finding any DNA segments that resemble potential spacer regions of cyanobacterial strains in the ten virtual cyanophages. In fact, the CRISPR-Cas arrays have not been well annotated in the 626 sequenced cyanobacterial genomes deposited in the CyanoOmics database (http://www.cyanoomics.cn), and moreover, it was reported that only ~40% of bacteria encode a CRISPR system [85]. An alternative approach is to hunt signature genes (usually refer to auxiliary metabolic genes) shared by both hosts and phages, such as *whiB* in *Actinobacteria* and their phages [86], *phoH* in marine phages and their hosts [87], or photosynthetic genes in cyanobacteria and cyanophages [88, 89]. Due to the limited information of cyanophages and corresponding hosts, we did not succeed in identifying host-derived auxiliary metabolic genes in the ten virtual cyanophages from the metagenomic data of the Lake Chaohu. Altogether, it is urgently needed to discover more reference genomes of both cyanophages and cyanobacteria.

## Conclusions

To better characterize the freshwater cyanophages, we isolated five cyanophages Pam1~Pam5 that infect a same strain of cyanobacterium *P. mucicola* Chao 1806 from the Lake Chaohu. Comparative genomic analyses revealed more than half of the ORFs encode proteins of unknown function, indicating plenty of “dark matter” in cyanophages. Using the present five and previously reported cyanophages as the reference, we developed a high-throughput strategy to systematically identify virtual freshwater cyanophages from the metagenomic data. Thanks to the increasing metagenomic data in combination with the rapid development of bioinformatic and artificial intelligence technologies, this strategy makes it possible to mine unculturable cyanophages, as well as bacteriophages and viruses. Subsequent whole-genome synthesis combined with microbiological experiments will largely promote the isolation of *bona fide* cyanophages. An enriched library of freshwater cyanophages will lay a foundation on the potential applications of cyanophages to regulate the cyanobacterial blooms in the future.

## Supplementary Information

**Additional file 1: Fig. S1.** The morphology of host cyanobacterium *P. mucicola* Chao 1806. **Fig. S2.** PCR assays indicated that Pam1 and Pam5 always coexist in the crude lysate of a single plaque of infection. **Fig. S3.** The circular genomic maps of cyanophages **a**-**c** Pam3~Pam5, respectively. **Fig. S4.** Whole-genome alignment of Pam1 against Pam5 that performed by the software Mauve. **Fig. S5.** Structural analyses of lysogeny-associated proteins of Pam1 and Pam5. **Fig. S6.** Comparisons of other three host CRISPR spacer regions against DNA segments of Pam2 genome. **Fig. S7.** Cartoon representations of predicted structures of putative RBPs from Pam1, 2, 3, 5. **Fig. S8.** Metagenomic fragments recruitment analyses against the genomes of **a**-**e** Pam1~Pam5 via RBB strategy, respectively. **Fig. S9.** The genomic maps of three virtual cyanophages **a**-**c** k141_145115, k141_145220 and k141_53315, respectively. **Fig. S10.** Multiple whole-genome alignments of the clustered **a** circular and **b** linear contigs with their neighboring cyanophages via software Mauve, respectively. **Table S1.** The coordinates of 11 estuaries that are major rivers to the Lake Chaohu. **Table S2.** Predicted ORFs of cyanophages Pam1~Pam5. **Table S3.** Four tRNA genes in the Pam2 genome. **Table S4.** Primary sequence similarities of the putative RBPs of cyanophages Pam1, 2, 3, 5. **Table S5.** DALI search results of the putative RBPs of cyanophages Pam1, 2, 3, 5. **Table S6.** Seven circular contigs assembled from the metagenomic data of the Nanfei estuary in October, 2017.

## Data Availability

The genomes of cyanophages Pam1~Pam5 reported in this study have been deposited in the GenBank database with the accession numbers of ON014753~ON014757, respectively. The raw viral metagenomic sequencing data reported in this study have been deposited in the Sequence Read Archive (SRA) database under the accession number of PRJNA817340. All data generated or analyzed during this study are included in this published article and its supplementary information files.
