# Supplementary material for: Comparative genomic analysis of five freshwater cyanophages and reference-guided metagenomic data mining
Source: Microbiome. 2022 Aug 17;10:128. doi: 10.1186/s40168-022-01324-w (PMC9382816; doi:10.1186/s40168-022-01324-w)
Supplement: Supplementary file 2 — Additional file 1: Fig. S1. The morphology of host cyanobacterium P. mucicola Chao 1806. Fig. S2. PCR assays indicated that Pam1 and Pam5 always coexist in the crude lysate of a single plaque of infection. Fig. S3. The circular genomic maps of cyanophages a-c Pam3~Pam5, respectively. Fig. S4. Whole-genome alignment of Pam1 against Pam5 that performed by the software Mauve. Fig. S5. Structural analyses of lysogeny-associated proteins of Pam1 and Pam5. Fig. S6. Comparisons of other three host CRISPR spacer regions against DNA segments of Pam2 genome. Fig. S7. Cartoon representations of predicted structures of putative RBPs from Pam1, 2, 3, 5. Fig. S8. Metagenomic fragments recruitment analyses against the genomes of a-e Pam1~Pam5 via RBB strategy, respectively. Fig. S9. The genomic maps of three virtual cyanophages a-c k141_145115, k141_145220 and k141_53315, respectively. Fig. S10. Multiple whole-genome alignments of the clustered a circular and b linear contigs with their neighboring cyanophages via software Mauve, respectively. Table S1. The coordinates of 11 estuaries that are major rivers to the Lake Chaohu. Table S2. Predicted ORFs of cyanophages Pam1~Pam5. Table S3. Four tRNA genes in the Pam2 genome. Table S4. Primary sequence similarities of the putative RBPs of cyanophages Pam1, 2, 3, 5. Table S5. DALI search results of the putative RBPs of cyanophages Pam1, 2, 3, 5. Table S6. Seven circular contigs assembled from the metagenomic data of the Nanfei estuary in October, 2017. [file 40168_2022_1324_MOESM1_ESM.zip › Supplementary Information20220509.docx]

**Supporting information**

**Supplementary figures and figure legends**


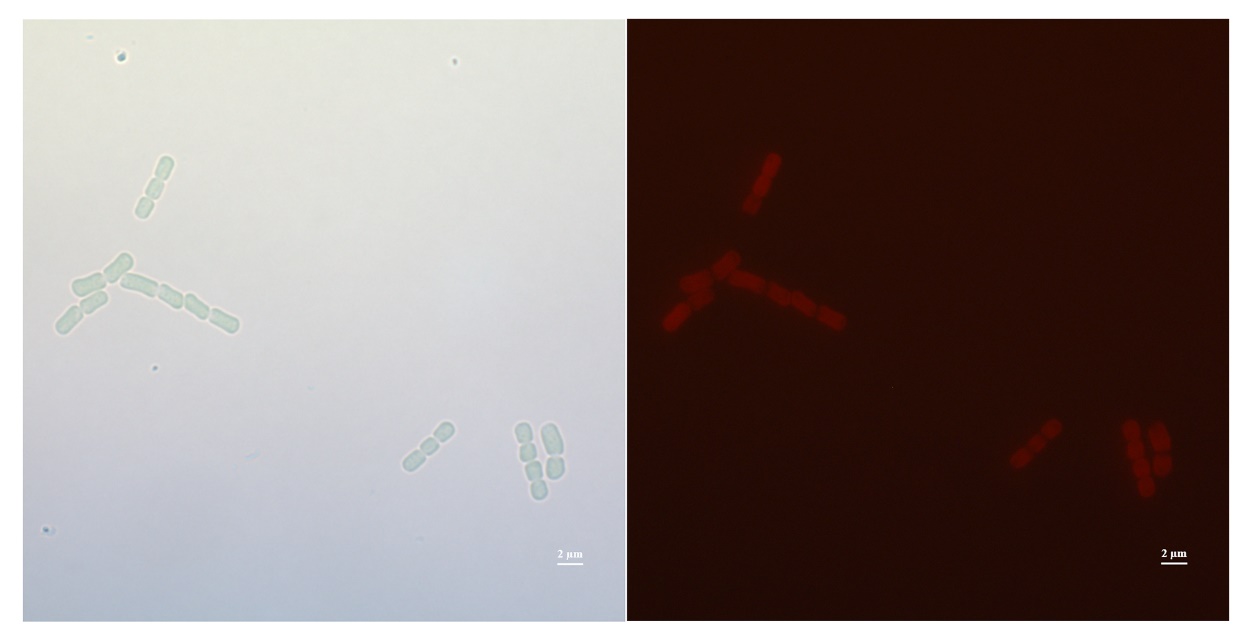


**Fig. S1** The morphology of host cyanobacterium *P. mucicola* Chao 1806. The photo was taken by an Axio Vert.A1 inverted fluorescence microscope (ZEISS). The scale bar is 2 μm.


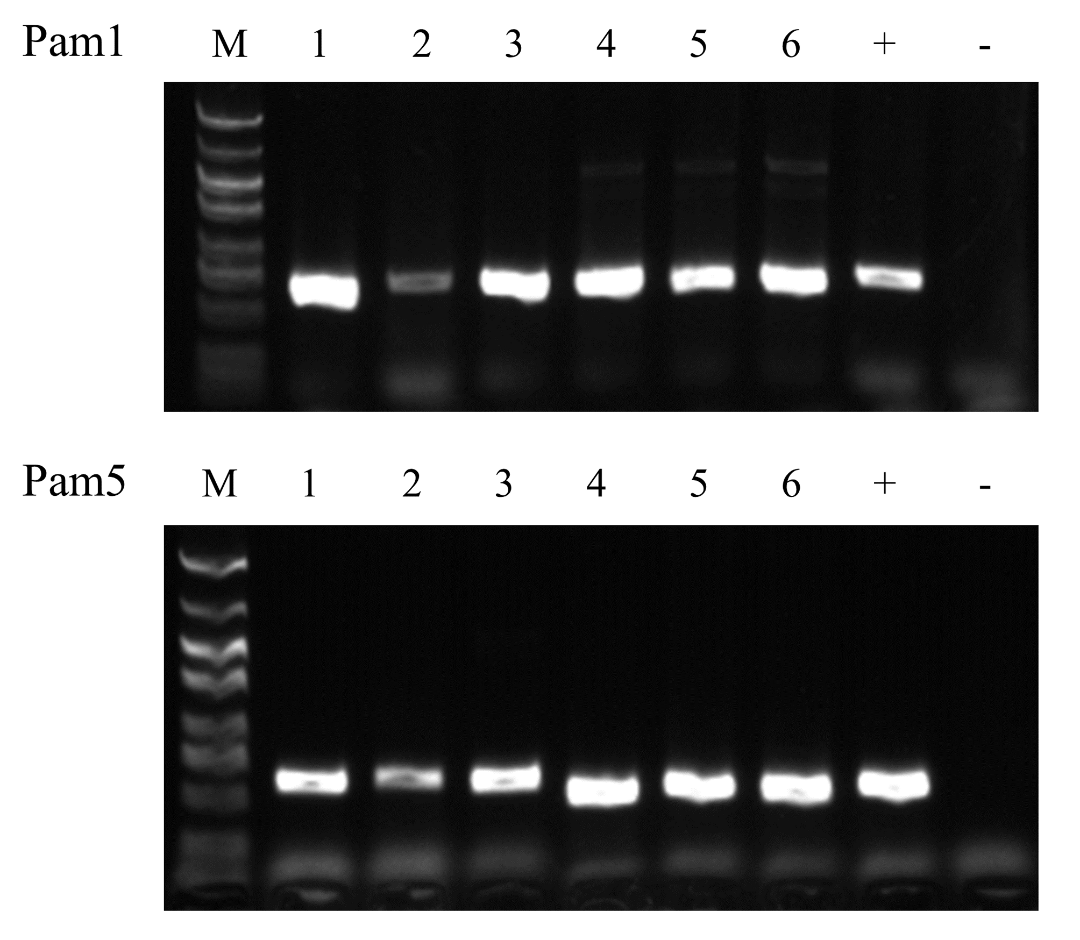


**Fig. S2** PCR assays indicated that Pam1 and Pam5 always coexist in the crude lysate of a single plaque of infection. M represents the DNA marker. “+” represents the positive control, whereas “-” represents the negative control. The numbers 1~6 mean the crude lysates of six single plaques, respectively.

**
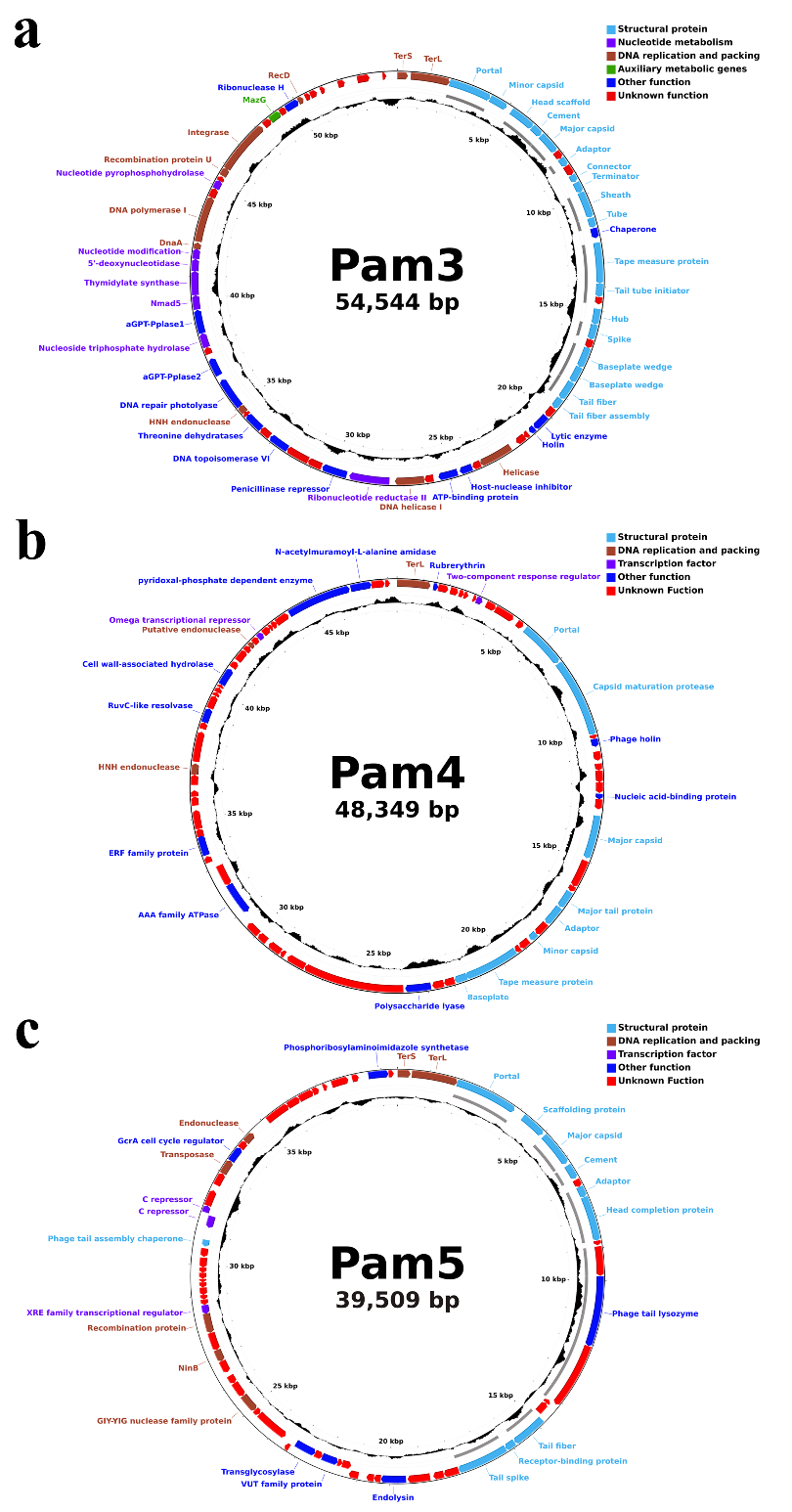
**

**Fig. S3** The circular genomic maps of cyanophages **a-c** Pam3~Pam5, respectively. Circles from the outmost to the innermost represent: predicted ORFs with known functions on (i) forward strand and (ii) reverse strand are labeled and colored based on their functions; (iii) structural proteins that identified by mass spectrometry are showed by gray lines; (iv) G+C content plotted relative to the genomic mean of 35% G+C.


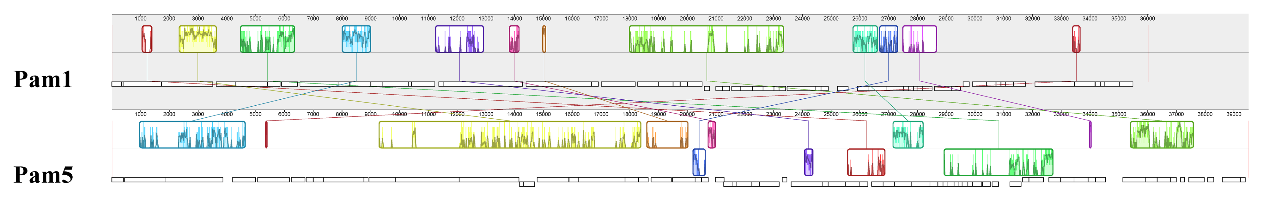
**Fig. S4** Whole-genome alignment of Pam1 against Pam5 that performed by the software Mauve. Blocks with the same color indicate the homologous regions of two genomes, which are also connected by the same color lines. The height of the similarity profile corresponds to the sequence similarity, whereas regions outside the colored blocks indicate the lack of homology among the two genomes. The inverted blocks are indicated below the genome’s center axis (a horizontal line). The numbers above the alignments indicate the nucleotide positions in the genome.


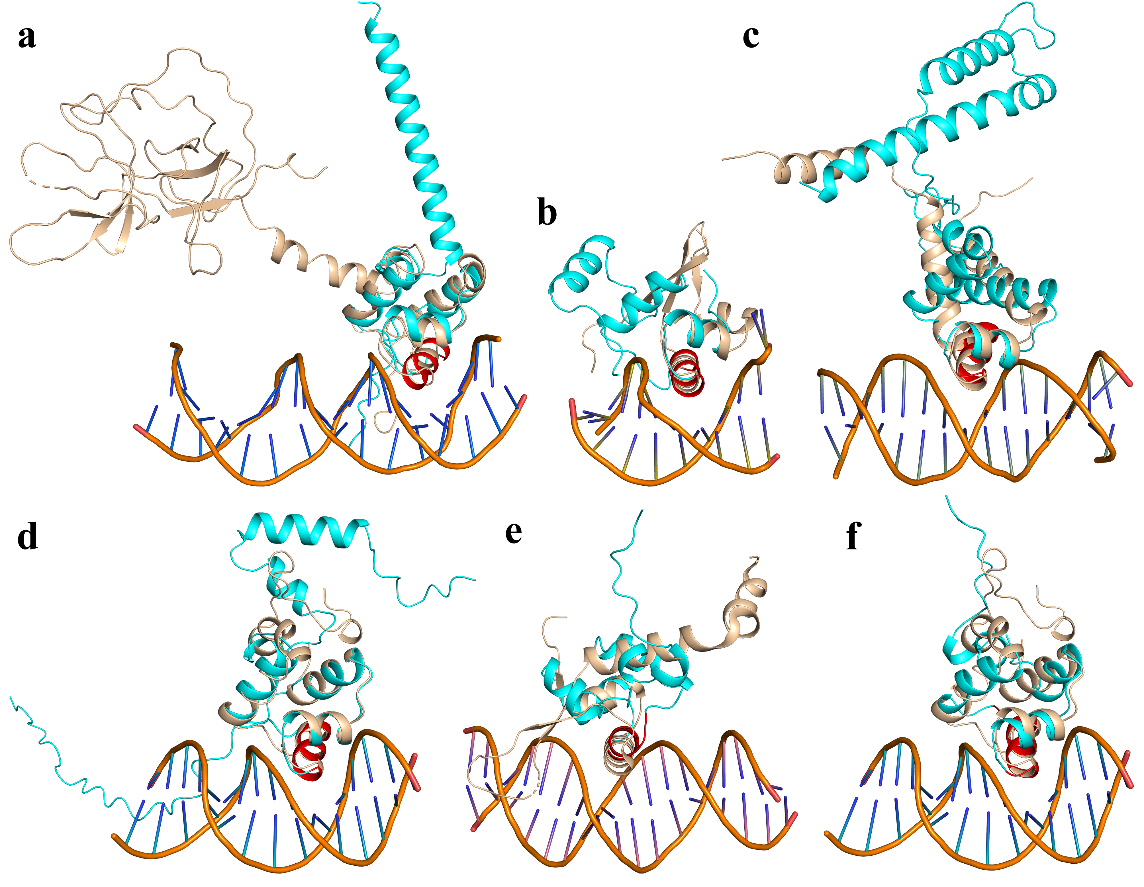


**Fig. S5** Structural analyses of lysogeny-associated proteins of Pam1 and Pam5. The structural comparisons of **a** gp47 against λ CI repressor, **b** gp48 against λ Cro, **c** gp49 against λ CII regulator, **d** gp52 against P2 C repressor, **e** gp53 against P2 Cox, and **f** gp53 against P2 C repressor were all done by CCP4i. The 3-D structure predictions of Pam1 gp47~gp49 and Pam5 gp52~gp53 were performed by AlphaFold 2.0, whereas the DNA-complexed structures of λ CI repressor (PDB: 3bdn), Cro (PDB: 6cro), CII regulator (PDB: 1zs4), together with apo-form structures of P2 C repressor (PDB: 2xcj) and Cox (PDB: 4hlf) were downloaded from the PDB database (https://www.rcsb.org/). The DNA-complexed structures of P2 C repressor and Cox were obtained by superposing against the DNA bound structures of P22 C2 (PDB: 2r1j) and ^λΔ^55Xis (PDB: 2og0), respectively. The proteins of Pam4 and Pam5 are colored in cyan, whereas the proteins of λ and P2 are colored in wheat. The DNA recognition helix is colored in red.


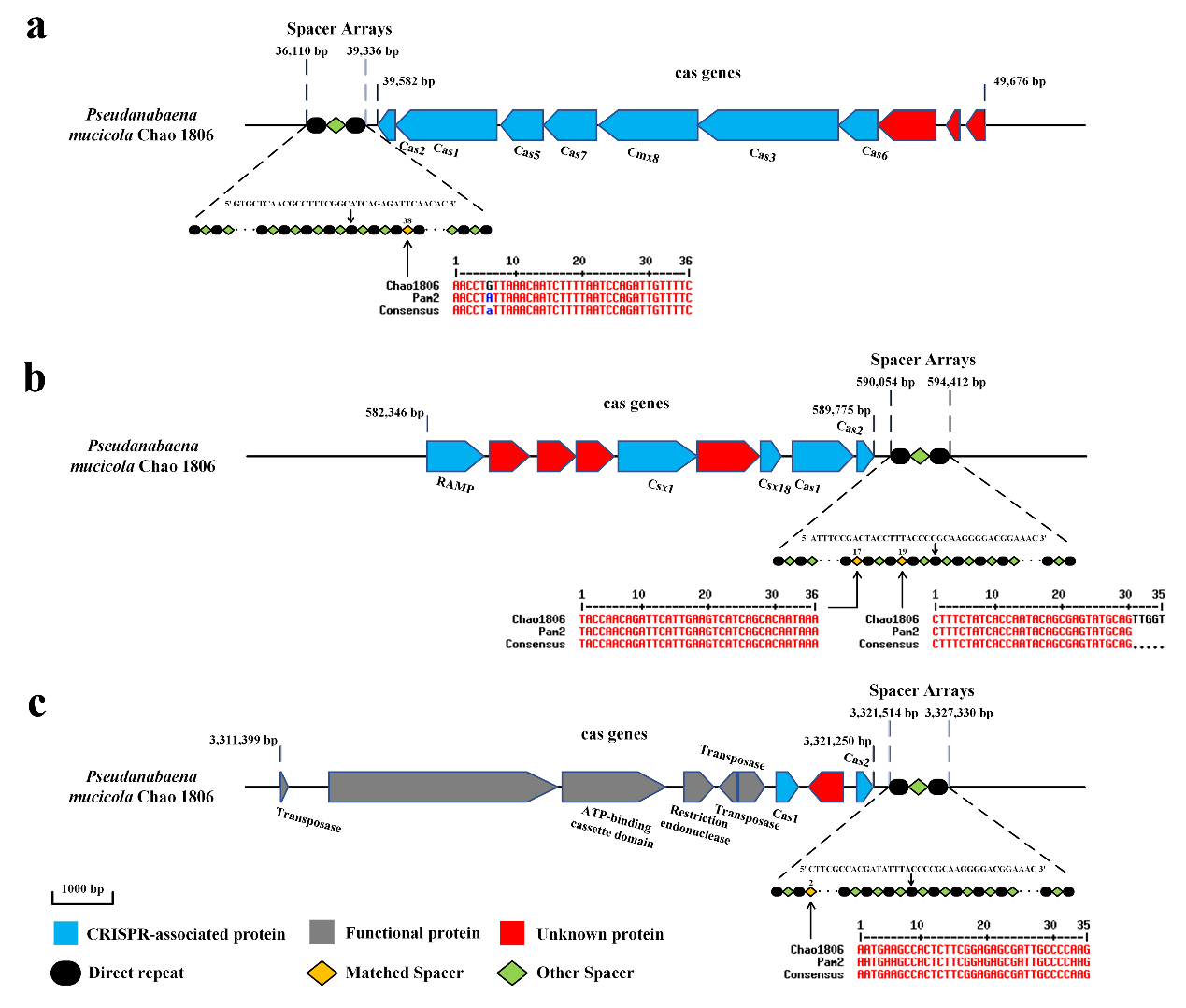


**Fig. S6** Comparisons of other three host CRISPR spacer regions against DNA segments of Pam2 genome. The alignments were performed by Multalin (http://multalin.toulouse.inra.fr/multalin/).


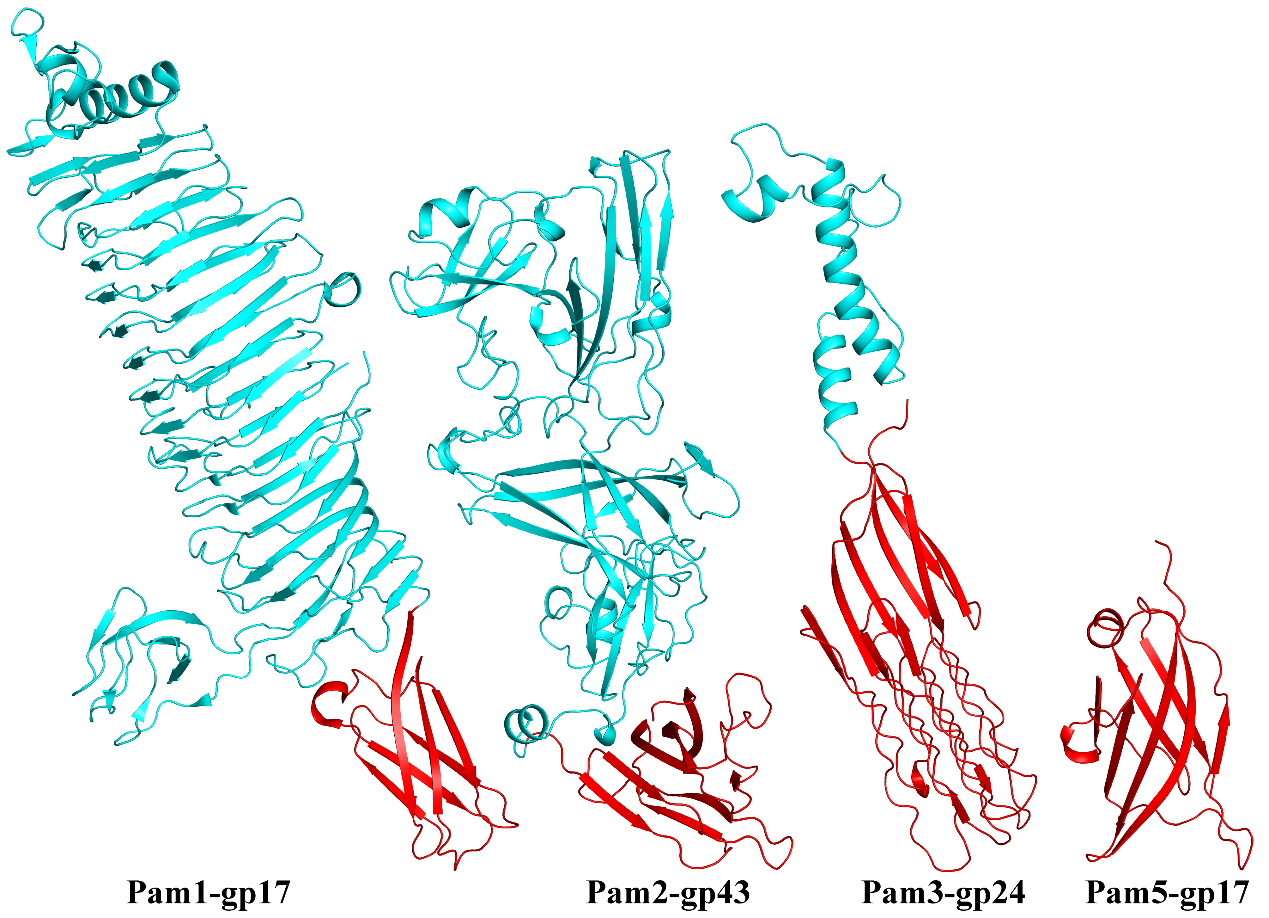


**Fig. S7** Cartoon representations of predicted structures of putative RBPs from Pam1, 2, 3, 5. The structural predictions were performed by AlphaFold 2.0. The N-terminal and C-terminal domains were colored in cyan and red, respectively.


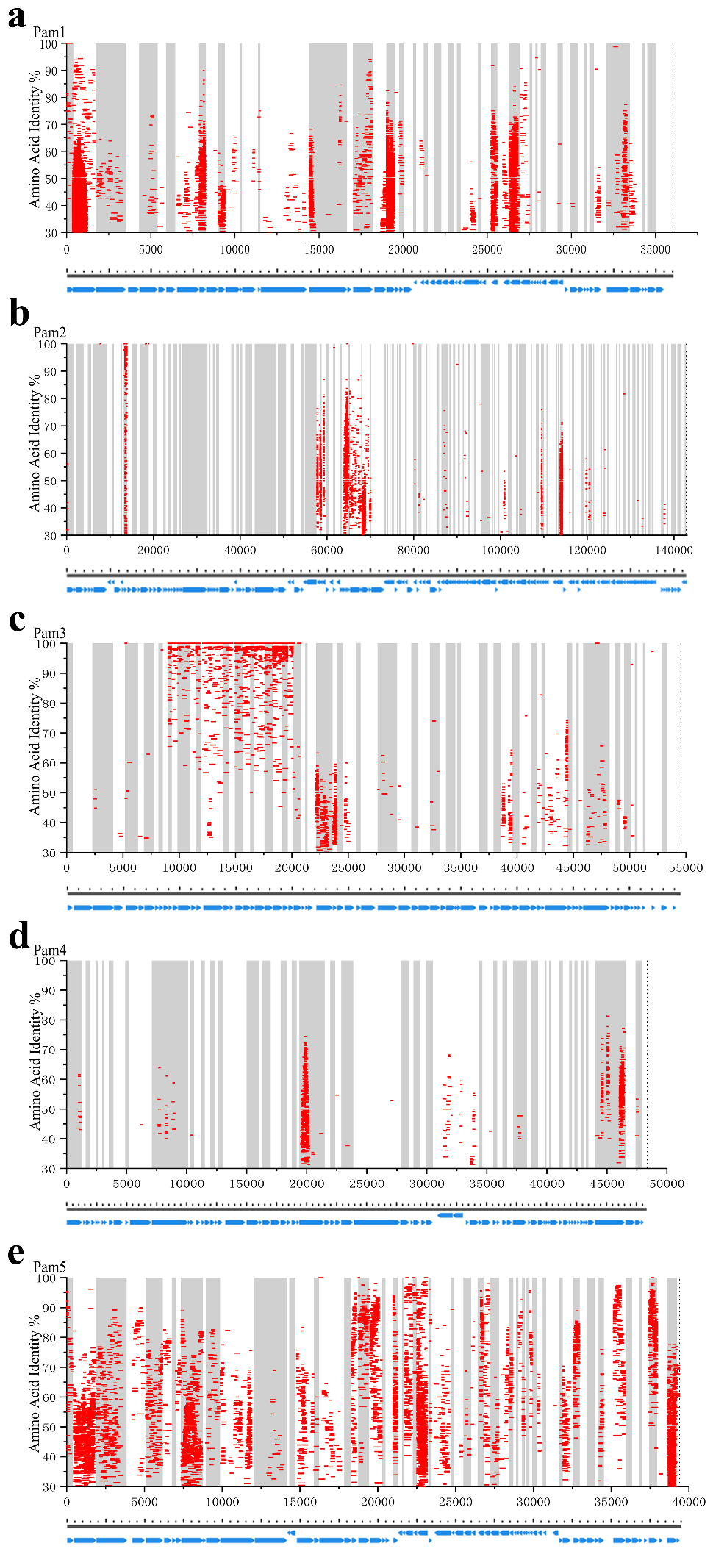


**Fig. S8** Metagenomic fragments recruitment analyses against the genomes of **a**-**e** Pam1~Pam5 via RBB strategy, respectively. Each red horizontal line represents a read recruited from the metagenomic data. The *X*-axis represents the genome length, whereas the *Y*-axis corresponds to the amino-acid identity of each read compared with that of Pam1~Pam5. The alternate white and grey background indicate the ORFs of each cyanophage, which are also displayed as blue arrows at the bottom of each panel.


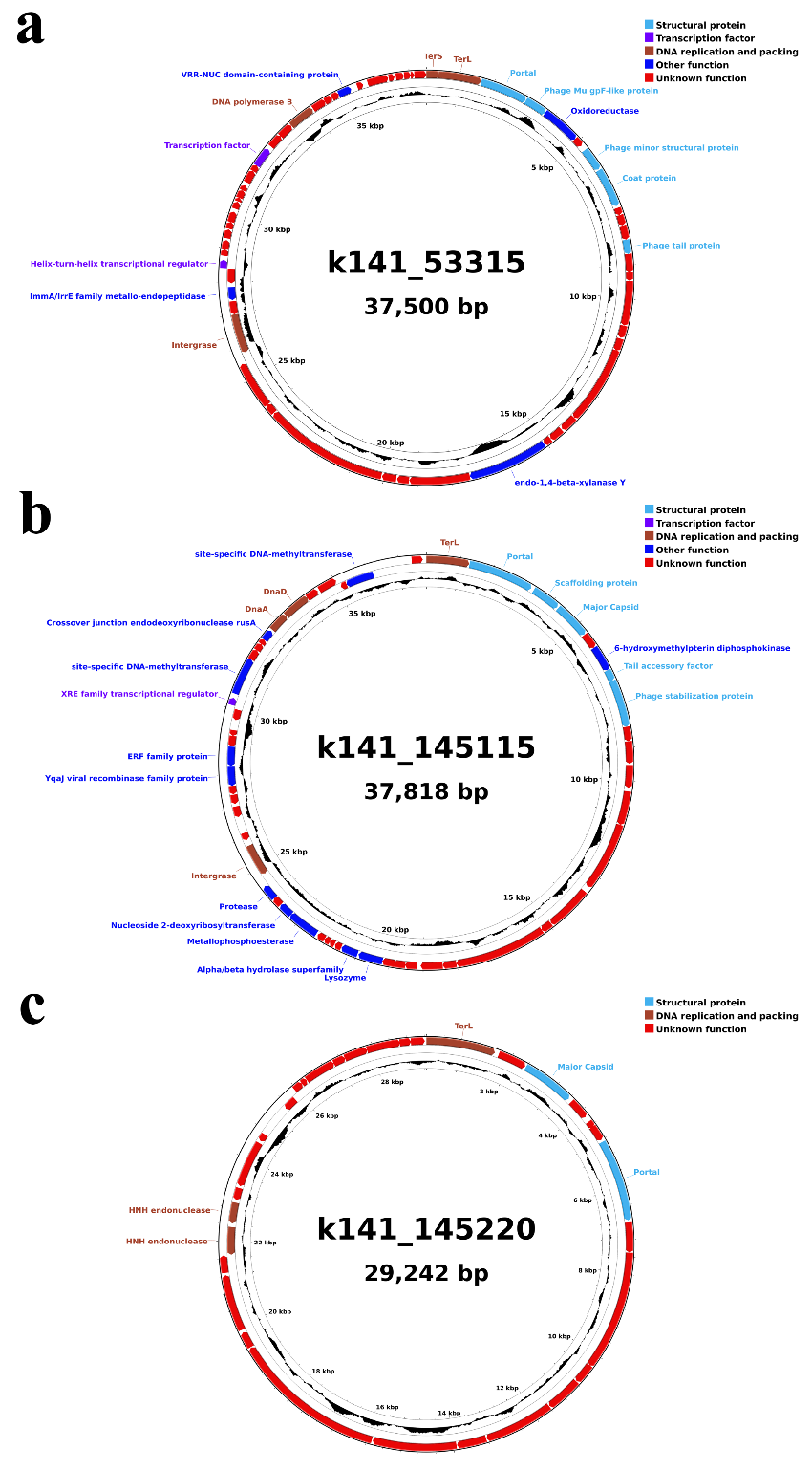


**Fig. S9** The genomic maps of three virtual cyanophages **a-c** k141_145115, k141_145220 and k141_53315, respectively. Circles from the outmost to the innermost represent: predicted ORFs with known functions on (i) forward strand and (ii) reverse strand are labeled and colored based on their functions; (iii) structural proteins that identified by mass spectrometry are showed by gray lines; (iv) G+C content plotted relative to the genomic mean of 35% G+C.


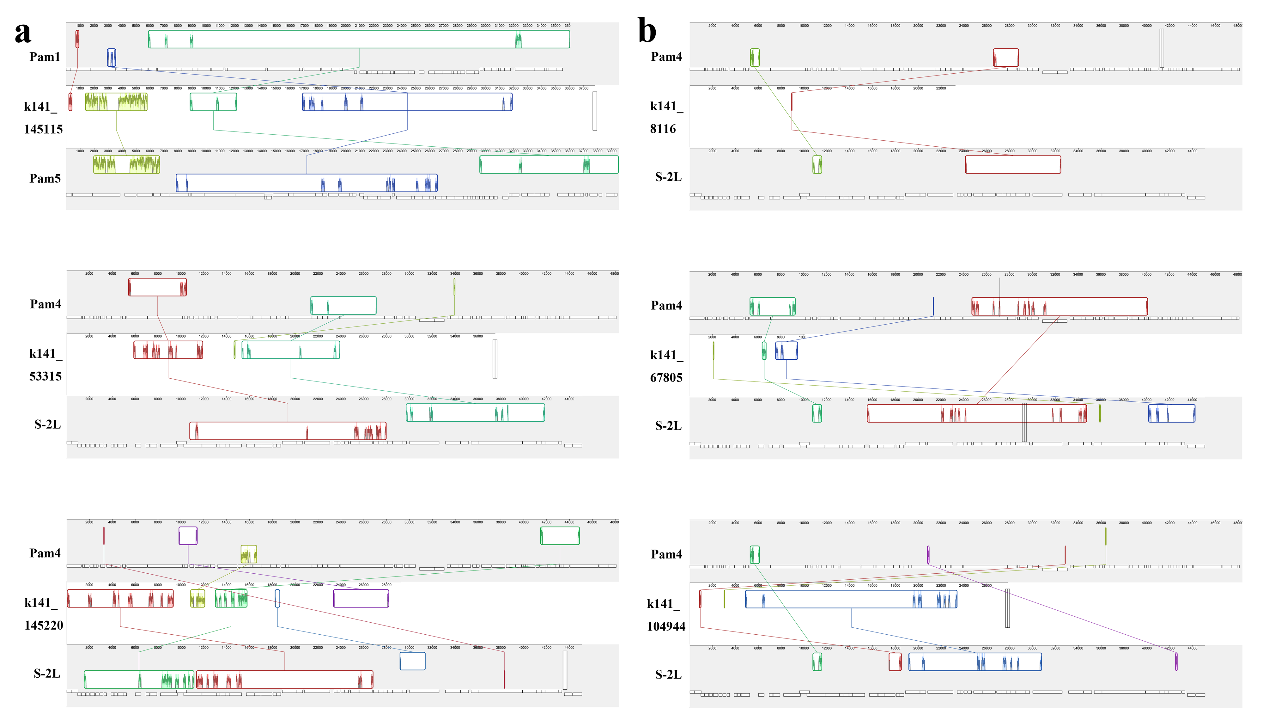


**Fig. S10** Multiple whole-genome alignments of the clustered **a** circular and **b** linear contigs with their neighboring cyanophages via software Mauve, respectively. Blocks with the same color indicate the homologous regions of two genomes, which are also connected by the same color lines. The height of the similarity profile corresponds to the sequence similarity, whereas regions outside the colored blocks indicate the lack of homology among the two genomes. The numbers above the alignments indicate the nucleotide positions in the genome.

**Supplementary tables**

**Table S1.** The coordinates of 11 estuaries that are major rivers to the Lake Chaohu.

| **Estuary** | **Latitude (N)** | **Longitude (E)** |
| --- | --- | --- |
| Shiwuli | 31°42′29.9′′ | 117°19′31.8′′ |
| Nanfei | 31°42′29.9′′ | 117°24′52.4′′ |
| Tongyang | 31°39′26.6′′ | 117°38′17.1′′ |
| Jiyu | 31°39′41.3′′ | 117°40′47.7′′ |
| Zhegao | 31°37′14.6′′ | 117°47′31.0′′ |
| Shuangqiao | 31°36′12.5′′ | 117°49′45.9′′ |
| Yuxi | 31°34′55.7′′ | 117°51′17.1′′ |
| Zhao | 31°25′26.8′′ | 117°33′0.3′′ |
| Baishitian | 31°31′47.5′′ | 117°23′55.3′′ |
| Hangbu | 31°32′56.3′′ | 117°21′24.0′′ |
| Xiapai | 31°39′59.5′′ | 117°17′28.5′′ |

**Table S3.** Four tRNA genes in the Pam2 genome.

| **tRNA number** | **tRNA start** | **tRNA end** | **tRNA type** | **tRNA type** |
| --- | --- | --- | --- | --- |
| 1 | 50607 | 50683 | Arg | TCT |
| 2 | 50686 | 50761 | Lys | TTT |
| 3 | 50765 | 50837 | Asn | GTT |
| 4 | 50841 | 50912 | Gly | TCC |

**Table S4.** Primary sequence similarities of the putative RBPs of cyanophages Pam1, 2, 3, 5.

|  | **Pam1-gp17** | **Pam2-gp43** | **Pam3-gp24** | **Pam5-gp17** |
| --- | --- | --- | --- | --- |
| **Pam1-gp17** | - | 16% | 12% | 5% |
| **Pam2-gp43** | - | - | 13% | 7% |
| **Pam3-gp24** | - | - | - | 12% |
| **Pam5-gp17** | - | - | - | - |

**Table S5.** DALI search results of the putative RBPs of cyanophages Pam1, 2, 3, 5.

| **Cyanophage** | **ORFs** | **Z-score** | **RMSD** | **Protein** | **PDB code** |
| --- | --- | --- | --- | --- | --- |
| Pam1 | gp17 | 29.6 | 2.7 | alpha-1,3-glucanase | 6k0v |
|  |  | 27.4 | 2.4 | glycoside hydrolase family 110 | 7jwf |
|  |  | 27.2 | 2.0 | cell wall surface anchor family protein | 3zpp |
| Pam2 | gp43 | 8.7 | 5.4 | particle-associated glycoside hydrolase | 6c72 |
|  |  | 8.0 | 13.8 | exopolysaccharide biosynthesis protein | 6tgf |
| Pam3 | gp24 | 4.6 | 16.3 | alpha-glucosidase | 5hqb |
| Pam5 | gp17 | 8.6 | 2.6 | cell surface glycoprotein | 7ptp |
|  |  | 8.5 | 2.9 | sugar ABC transporter substrate-binding protein | 6xds |

**Table S6.** Seven circular contigs assembled from the metagenomic data of the Nanfei estuary in October, 2017.

| **Seq_name** | **Contig_length (bp)** | **Hallmark_cnt** | **Group** | **Shape** |
| --- | --- | --- | --- | --- |
| k141_8298 | 34,014 | 6 | dsDNA phage | circular |
| k141_53315 | 37,500 | 4 | dsDNA phage | circular |
| k141_65279 | 43,018 | 6 | dsDNA phage | circular |
| k141_117671 | 41,765 | 3 | dsDNA phage | circular |
| k141_145109 | 32,740 | 5 | dsDNA phage | circular |
| k141_145115 | 37,818 | 5 | dsDNA phage | circular |
| k141_145220 | 29,242 | 2 | dsDNA phage | circular |
